# 2154. Cross-resistance of Ceftolozane-Tazobactam and Imipenem-Relebactam Against Clinical *P. aeruginosa* Isolates from Bloodstream and Respiratory Tract Infections– SMART United States 2019-2021

**DOI:** 10.1093/ofid/ofad500.1777

**Published:** 2023-11-27

**Authors:** Sibylle Lob, Mark G Wise, Karri Bauer, Fakhar Siddiqui, David W Hilbert, John Esterly, C Andrew DeRyke, Katherine Young, Mary Motyl, Daniel F Sahm

**Affiliations:** Merck & Co., Inc., Schaumburg, Illinois; IHMA, Schaumburg, Illinois; Merck & Co., Inc., Schaumburg, Illinois; Merck & Co., Inc., Schaumburg, Illinois; Merck Research Laboratories, Rahway, New Jersey; Merck & Co., Inc., Schaumburg, Illinois; Merck & Co., Inc., Schaumburg, Illinois; Merck, Rahway, New Jersey; Merck, Rahway, New Jersey; IHMA, Schaumburg, Illinois

## Abstract

**Background:**

Antimicrobial resistance among *Pseudomonas aeruginosa* is challenging with limited treatment options. Ceftolozane/tazobactam (C/T) maintains activity against *P. aeruginosa* resistant to commonly used β-lactams. Imipenem/relebactam (IMI/REL) can restore the activity of IMI against *P. aeruginosa*. C/T and IMI/REL can be affected by different mechanisms of resistance, and pathogens can be nonsusceptible to one agent but susceptible to the other. We evaluated the activity of C/T and IMI/REL against *P. aeruginosa* isolates collected from patients with lower respiratory tract (LRTI) and bloodstream (BSI) infections in the United States as part of the global SMART surveillance program.

**Methods:**

In 2019-2021, 24 US clinical labs collected up to 100 consecutive, aerobic or facultative, gram-negative pathogens from LRTI and up to 50 from BSI. Of 8643 collected isolates, 1986 (23%) were *P. aeruginosa*. Susceptibility was determined with CLSI broth microdilution and 2023 CLSI breakpoints.

**Results:**

Among *P. aeruginosa* BSI and LRTI isolates, 88% were susceptible (S) to both C/T and IMI/REL, 2% were nonsusceptible (NS) to both agents; 8% were S to C/T but not to IMI/REL, and 2% were S to IMI/REL but not to C/T (Table 1). Among MDR and DTR isolates, 45% and 29%, respectively, were S to both C/T and IMI/REL and 11% and 20%, respectively, were NS to both. Among C/T-NS isolates, 57.3% and 44.0% were S to IMI/REL and ceftazidime/avibactam (CZA), respectively (Table 2). Among IMI/REL-NS isolates, 83% were C/T-S, 69% CZA-S, and < 43% were S to all other studied β-lactams and LVX. Among CZA-R isolates, 61% and 47% remained S to C/T and IMI/REL, respectively.

**Figure:**
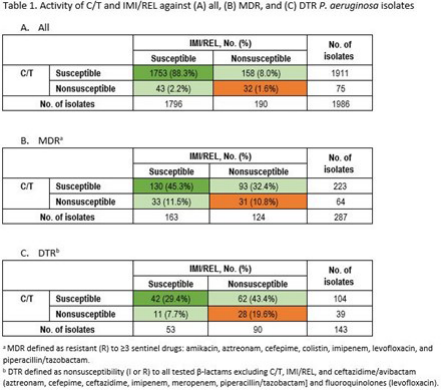

**Figure:**
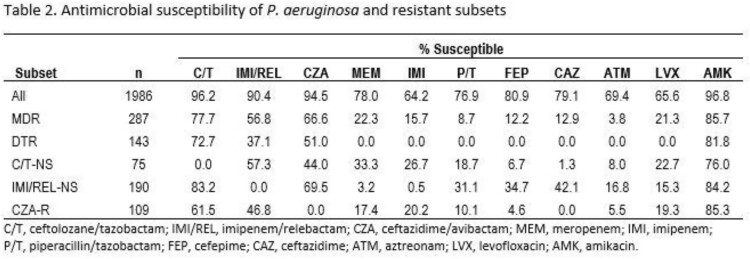

**Conclusion:**

Resistance to both C/T and IMI/REL was not common among recent clinical isolates of *P. aeruginosa* from the US, and both agents represent important treatment options. A significant proportion of isolates NS to one agent were S to the other, especially among MDR and DTR isolates. The data suggest that susceptibility to both agents should be tested.

**Disclosures:**

**Sibylle Lob, MD**, Merck & Co., Inc.: Honoraria **Mark G Wise, PhD**, Merck & Co., Inc.: Honoraria|Pfizer Inc.: Honoraria|Venatorx: Paid fees for conducting the study and abstract preparation **Fakhar Siddiqui, MD, MBA**, Merck & Co Inc.: Employee **Daniel F. Sahm, PhD**, Merck & Co., Inc.: Honoraria|Pfizer Inc.: Honoraria|Venatorx: Paid fees for conducting the study and abstract preparation

